# Campus activism in polarized times: Amy J. Binder and Jeffrey L. Kidder, *The Channels of Student Activism: How the Left and Right Are Winning (and Losing) in Campus Politics Today*, University of Chicago Press, 2022

**DOI:** 10.1007/s10734-023-01037-5

**Published:** 2023-05-01

**Authors:** Daniel Meyer

**Affiliations:** Department of Education and Social Sciences, University of Cologne, Gronewaldstr. 2, 50931 Cologne, Germany

Universities are part and parcel of the divisions in contemporary American politics. Controversies over free speech, identity politics, and cancel culture rock college campuses across the United States (e.g., Ben-Porath, 2023; Chemerinsky & Gillman, 2017; Palfrey, 2017), echoing the political correctness debates of the 1990s (see Berman, 1992). The boundaries between free expression and hate speech, the disinvitation of controversial speakers, and the need for safe spaces and trigger warnings are some of the most fiercely debated issues. Although party lines are not as clear as one might expect, conservatives generally accuse overly liberal academic institutions of indoctrinating and coddling students, while progressives tend to emphasize the experiences of minority students and advocate for a more inclusive campus environment.

In *The Channels of Student Activism*, Amy Binder and Jeffrey Kidder take us to the front lines of America’s culture war by exploring political polarization through the lens of student activism. The book offers a rich, detailed analysis of the cultural and organizational mechanisms that channel students through distinctive pathways which create “the contours of contemporary campus politics” (p. 9). On the one hand, conservative students are pulled outside by right-leaning organizations, which support them with financial resources and offer students career opportunities outside academia, while on the other hand, progressive students are drawn inside their universities through their close ties with student affairs offices, multicultural centers, and academic departments.

In theoretical terms, the book draws upon a culturally informed organizational perspective (e.g., Armstrong & Hamilton, 2013; Kaufman & Feldman, 2004; Reyes, 2018) and could be read as an extension of the authors’ past work on conservative students (see Binder & Wood, 2013; Kidder & Binder, 2020). This line of research investigates how students navigate through college and become socialized on campus. The assumption underlying this literature is that universities have the power to transform students well beyond the processes of professionalization and to shape students’ racial, sexual, or political identities. The book contributes to this debate by convincingly demonstrating how activists are being sorted and shaped by political clubs and channeled along different political paths during college. While past research has focused on either progressive or conservative students, this book is among the first to discuss the entire political spectrum and to identify distinctive organizational mechanisms; specifically, Binder and Kidder’s outside-in/inside-out model offers a thorough and compelling framework to understand current campus controversies.

Methodologically, the study is based on 77 semi-structured interviews with politically engaged college students from the left, center, and right enrolled at four different public universities across the United States during the 2017–2018 academic year. The authors also spoke with 21 political organization leaders and 16 faculty and staff members. In contrast to Amy Binder and Kate Wood’s 2013 book *Becoming Right*, the different universities were not selected because of their different political styles, but due to their commonality: all universities were located in battleground states. This is important to mention because the study is not representative of the general—mostly apolitical and apathetic—studentship, but purposefully oversampled politically engaged students in highly politicized environments. While this misrepresentation may not concern those who are interested in the dynamics of student activism at the front lines, it might bother those seeking to understand how divided college campuses truly are. The interviews are complemented by survey data from the CIRP Freshman Survey, which is presented in chapter 2, and other sources, such as firsthand observations and college club social media posts, providing a well-rounded examination of student activism. Notably, a stronger focus on ethnographic data would have been useful; I will revisit this point later.

The book is divided into seven chapters, beginning with a beautifully written introduction that transports the reader to the front lines of student activism and ends with a general overview of the book. Chapter 2, jointly written with Ellen Stolzenberg, contributes a discussion of national trends in students’ political views by subdividing the political spectrum into three main groups: progressives, made up of leftists and liberals; conservatives, made up of conservatives and libertarians; and moderates. In this chapter, we learn that while students who identify as middle-of-the-road make up the majority, the share of liberals has increased and the proportion of conservatives has remained virtually the same over the past 15 years. This chapter introduces readers to American students—both in terms of their socio-demographic backgrounds and to their different positions, from an orientation toward social justice, queer liberation, and anti-capitalism on the left to concerns about freedom of speech and immigration on the right; the ambivalent positions of many libertarians; and increasing tensions within conservatism, especially between traditionalists and populists. Again, it should be noted that even though moderate students are the majority on campus, they are systematically underrepresented in the study, “likely because they are less inclined to get involved in politically active clubs” (p. 20).

Student-led groups are the focus of chapter 3. Here Binder and Kidder describe the actual process of political mobilization and organizational socialization. Using the well-known metaphors of sieves and incubators, they illustrate how political clubs sort and shape students’ distinctive activism styles. Through clubs, “classmates are transformed into opponents or potential collaborators” (p. 49). One strength of the book is its close attention to membership recruitment processes, activism styles, and the political tactics cultivated in different clubs. According to the authors, progressive clubs tend to target the school’s administration, while conservative clubs tend to target the overall campus culture. For example, leftist groups often make direct policy demands of administrators, such as compulsory sensitivity training for incoming freshmen or the removal of monuments they consider offensive, whereas students on the right aim to disrupt the dominant progressive discourse found at their schools by hosting conservative speakers with alternative viewpoints.

In the following two chapters, *The Channels of Student Activism* abstracts from individual student clubs and presents two major student activism channels: the conservative channel and the progressive channel. By analyzing the mechanisms of three conservative organizations—the American Enterprise Institute, Turning Point USA, and PragerU—chapter 4 highlights the generous outside support available for right-leaning students, ranging from financial resources for campus recruiting, conference travel, and summer fellowships to opportunities to bring speakers onto campus and templates for media blitzes. Binder and Kidder make it clear that conservative organizations not only channel activism, but also create a career funnel that helps young activists find paid internships and occupational opportunities within the conservative sector. In contrast, students in the progressive channel are unable to build upon external sponsorships, but must navigate in more of a do-it-yourself environment, both in terms of political activism and future career prospects. Yet, left-leaning students benefit from being embedded within their universities. During college, they meet like-minded peers, are instructed by (mostly) liberal professors, and profit from various multicultural centers that cater to the needs of Asian-American, Black, and LGBTQ students. While liberal students often experience a deep sense of belonging on campus, activists on the far left are frequently disappointed by what they perceive as sluggish efforts by the university staff to promote an inclusive environment. As Binder and Kidder explain: “Ironically, such close ties can blind progressives to the myriad ways their ideological orientations are actually supported day in and day out at their schools. Fish are unaware of the water in which they swim” (p. 101).

The controversy over free speech is the subject of chapter 6, previously published in *Sociological Forum*. This chapter begins with a short discussion of different interpretations of the First Amendment, from absolutist to realist positions, and reminds us of the changing political orientation of free speech advocates—from the progressive Free Speech Movement of the 1960s to conservatives’ current “war on woke.” Binder and Kidder make clear that debates around free speech are highly polarized: While conservatives promote a largely unregulated marketplace of ideas, progressives aim to restrict certain types of what they deem to be hate speech. The chapter is at its best when it shows the microdynamics of action and reaction: Right-leaning students who feel muted on overly liberal campuses invite controversial speakers to bring in other viewpoints. At the same time, these provocative figures—whether intentionally or unintentionally—offend progressive students, who then attempt to silence or deplatform right-leaning speakers. What follows is conservative outrage over cancel culture on campuses, which again evokes for a reaction from progressives, and a reinforcing spiral of outrage is created, which is what has ultimately changed the fundamental culture of political debates.

The authors should be praised for their decision to not leave readers feeling discouraged, but to conclude their book with a chapter that encourages cross-partisan dialogue. By discussing the role of trans-partisan dialogue groups—groups that aim to build bridges between conservative and progressive groups—Binder and Kidder implicitly call for a better debate culture that respects diverse viewpoints and may prove to be more empathetic to the “deep stories” (Hochschild, 2016) of the other side. While there is a risk of stagnant, unproductive dialogues or the danger of being labeled a traitor, trans-partisan groups have the power to reconcile both sides and improve debate culture on campuses. What I am missing, however, is a discussion of how the logic of trans-partisan groups can be translated into permanent structures. One idea that came to my mind is the assignment of *advocatus* *diaboli* positions within every organizational unit to balance one-sided discussions. Another thing that the authors do not mention, but that is compatible with the concept of trans-partisan groups are systems of representation and self-governance, such as student councils or student unions, which are democratic institutions intended to foster deliberative discussions. A final point worth considering are university courses that aim to bring current campus controversies to the classroom and provide students a platform to discuss heated topics. At the University of Cologne, for example, we recently offered a graduate course on campus debates over identity politics and free speech that gave students the opportunity to reflect on their own campus environment.

When Binder and Kidder refer to organizations such as Heterodox Academy or position themselves as “champions” of the progressive project in higher education (p. 160), they make it clear that questions of partisanship go well beyond the studentship and affect the scholarly community as a whole. In other words, academics themselves are not objective observers, but are part of the field they study and therefore may have an own stance on issues surrounding free speech and academic freedom. In this sense, the book also speaks to the *homo academicus*. How higher education scholars can remain agnostic in an increasingly politicized field has been a long-debated question. One solution might be to recall what Max Weber ([1919] 1946) famously called “intellectual integrity” or what Pierre Bourdieu (2003) termed “participant objectivation,” both referring to an intellectual stance that asks researchers to reflect on their own socialization in academia and to push themselves to actively search for counterevidence. Another solution to the problem of objectivity in contested fields may lie in collaborative research projects. Daniel Kahneman (2003) coined the term “adversarial collaboration” and defined it as a collaboration of researchers with opposing viewpoints. For example, as a follow-up of the controversies surrounding Matthias Revers and Richard Traunmüller’s (2020) study on the politics of free speech at Goethe University Frankfurt, scientists with opposing views are now designing a pre-registered replication study to determine whether the results of the initial study hold (see Wuttke et al., 2021). In my view, initiatives like this could be an example and guide the work of other scholars working in highly contested fields such as the one upon which the reviewed book is premised.

There are two more points that warrant further discussion. The first concerns Binder and Kidder’s focus on interviews as their method of choice. Considering that the book draws on a “culturally informed organizational perspective” (p. 7) in the tradition of Elizabeth Armstrong and Laura Hamilton (2013) or Daisy Verduzco Reyes (2018), the organizational culture of political student clubs is not described as thickly as it could be. A possible reason for this shortcoming could be that the book relies too heavily on interviews and too little on ethnographic observations. As I have argued elsewhere (Meyer, 2022), it could prove to be useful to think of student clubs as distinctive settings within the larger ecology of a university and to pay close attention to the inner life and material culture of each organization. Furthermore, the authors could have traced students’ connections to other settings, such as student councils, career services, or fraternities, to provide a more complete picture of the cultural-organizational structure of campus politics. Questions the authors did not ask that could have provided a more holistic description of the organizational structure of campus activism include: What kinds of objects can be found in progressive clubs, and how do they differ from artifacts found in conservative clubs? How do activists behave in political clubs, and how does this differ from their behavior in other settings, such as lectures or dorms? Do students separate their different roles, or do they identify as full-time activists? How do career services help to advance or mitigate political careers? What is the relationship between student unions and extracurricular clubs? Do student governments conciliate the left and the right?

The second point concerns the temporal and regional generalizability of the study. Five years after data collection, American politics seems to be even more polarized. With COVID-19 and former President Donald Trump’s “stop the steal” campaign, new talking points have emerged that are accompanied by so-called alternative facts and conspiracy theories. It therefore remains an open question to what extent the last few years have impacted higher education and whether there have been any changes to the student activism channels analyzed in the book. While this does not make Binder and Kidder’s book outdated, an empirical update would be desirable. For example, conservatives, while still aiming to change campus culture, as the book analyzed, in the last years have also begun targeting school policies to try to ban books or propose free speech acts, a strategy Binder and Kidder assigned to progressive groups. Additionally, future research could compare different institutions, including public *and* private universities, community *and* liberal arts colleges, as well as institutions from different countries, since different universities are embedded in different organizational, cultural, and political structures. Other countries could be less polarized than the United States and therefore foster different forms of student activism. In Europe, for example, the relatively strong green parties might have shifted the focus of European activists more to issues of climate change, a topic that is virtually absent from the book.

Overall, *The Channels of Student Activism* is a valuable and insightful resource for anyone interested in student cultures, campus life, or collegiate activism, and a definite must-read for researchers working at the intersection of higher education, cultural, and political sociology. With their outside-in/inside-out approach, Binder and Kidder provide a useful framework to make sense of seemingly contradictory developments in student activism and offer an empirically nuanced contribution to current debates around political polarization, free speech, and identity politics. As the book is very well written and assumes no prior knowledge, it is also accessible to those who wish to better understand the political dynamics on college campuses and the impact thereof on the cultural divisions in contemporary societies, both in the United States and beyond.
